# Just preparation for war and AI-enabled weapons

**DOI:** 10.3389/fdata.2023.1020107

**Published:** 2023-05-12

**Authors:** Mitt Regan, Jovana Davidovic

**Affiliations:** ^1^Georgetown Law, Georgetown College, Georgetown University, Washington, DC, United States; ^2^Philosophy Department, The University of Iowa, Iowa City, IA, United States

**Keywords:** automated, weapons, testing, security, war

## Abstract

This paper maintains that the just war tradition provides a useful framework for analyzing ethical issues related to the development of weapons that incorporate artificial intelligence (AI), or “AI-enabled weapons.” While development of any weapon carries the risk of violations of jus ad bellum and jus in bello, AI-enabled weapons can pose distinctive risks of these violations. The article argues that developing AI-enabled weapons in accordance with jus ante bellum principles of just preparation for war can help minimize the risk of these violations. These principles impose two obligations. The first is that before deploying an AI-enabled weapon a state must rigorously test its safety and reliability, and conduct review of its ability to comply with international law. Second, a state must develop AI-enabled weapons in ways that minimize the likelihood that a security dilemma will arise, in which other states feel threatened by this development and hasten to deploy such weapons without sufficient testing and review. Ethical development of weapons that incorporate AI therefore requires that a state focus not only on its own activity, but on how that activity is perceived by other states.

## Introduction

Emerging attention to *jus ante bellum* as an element of the just war tradition reflects attention to “just preparation for war.” As Ned Dobos frames the issue, “When (if ever) and why (if at all) is it morally permissible to create and maintain the potential to wage war?” (Dobos, 2020, p. 2). We agree with Cecile Fabre that maintaining a standing army that is prepared to wage war if need be is morally justified because it enables a state to protect persons from violent infringements of their fundamental rights (Fabre, 2021). We argue, however that *jus ante bellum* still requires a state to morally justify the particular ways in which it engages in such preparation. Harry van der Linden suggests that this requires that a state prepare for war in ways that minimize the risk of unjust resort to force—violations of *jus ad bellum*—and unjust use of force during war—violations of *jus in bello* (van der Linden, 2010, p. 7).

This essay examines what *jus ante bellum* requires of states regarding the development and deployment of weapons enabled by artificial intelligence (AI). We define these as weapons that utilize artificial intelligence and machine-learning models in the targeting process, which may include tasks such as object recognition, target identification, or decision-support. We focus on the targeting process, and define AI-enabled weapon systems as those that use AI in that process, because the human-machine interactions in the targeting stages have the most consequential effects on war and on the ways in which norms of war may be violated (Ekelhof, 2018). To clarify, the targeting process consists of several steps at which humans and machines may interact in complex ways, with machines augmenting rather than displacing human judgment. But even when a human is the ultimate decision-maker at the last step, these interactions can shape their understanding of the situation they confront in powerful ways, which in turn influences their decision as to whether to fire.^1^

We believe that, in light of increasing attention by several states to the potential for incorporating AI into weapon systems, a state is justified in investing in developing such systems in order to protect its population [see Boulanin and Verbruggen (2017) for a discussion of the current state of such efforts]. We argue, however, that *jus ante bellum* requires that before deploying these weapons a state must engage in a rigorous testing, evaluation, verification, and validation (TEVV) process, which we describe below. It must also carefully consider the appropriate delegation of tasks between machines and humans.^2^ Finally, it must engage in development of these weapons in ways that do not trigger a security dilemma that leads other states to deploy AI-enabled systems without engaging in these processes.

These requirements reflect concern that premature deployment of AI-enabled weapon systems, and the deployment of systems with an inappropriate delegation of authority between machines and humans, increase the risk of violations of *jus ad bellum* and *jus in bello*. The next section elaborates on these risks.

## Risks of AI-enabled weapons

Aside from the risks that arise in any complex tightly coupled system, AI-enabled weapons have at least two features that can pose distinctive risks. First, systems at this point tend to be brittle, in the sense that they are not able to function effectively outside the specific set of circumstances for which they are programmed. It can be challenging for operators to identify when this happens, and to predict the consequences. Second, a system may not be able to provide an explanation of its analysis and recommendations in terms that are comprehensible to a human operator. This opacity can make it difficult for humans to exercise effective judgment about potential courses of action.^3^

These features of AI-enabled weapon systems could increase the risk of violations of *jus ad bellum* and *jus in bello*. With respect to *ad bellum*, states could field systems that are less flexible than conventional weapons and lack sensitive contextual awareness of likely human intentions. “This brittleness of machine decision-making may particularly be challenging in pre-conflict crisis situations, where tensions among nations run high,” and contextual human judgment can be crucial in lessening the risk of escalation (Horowitz and Scharre, 2021). Furthermore, even if a system performs as intended, adversaries may not know whether its behavior reflects human intention. This ambiguity may lead to escalation of conflict if states assume that they must ascribe hostile intention to an adversary in order to protect themselves.

With respect to *in bello* violations, delegation of some tasks to machines could mean that “minor tactical missteps or accidents that are part and parcel of military operations in the chaos and fog of war, including fratricide, civilian casualties, and poor military judgment, could spiral out of control and reach catastrophic proportions before humans have time to intervene” (Horowitz and Scharre, 2021). This risk would be exacerbated by the interaction between and among competing AI-enabled systems, which could result a cycle of attacks and counterattacks at a speed that humans could not control.

These risks underscore the crucial importance of rigorous pre-deployment review of AI-enabled weapons. States ordinarily would have incentives to engage in such review to ensure that they can exercise effective control of these weapons. Their willingness to do so could be lessened, however, by what is called the security dilemma. This occurs when states perceive that other states' military investment make them less secure, a perception that may be especially likely because of the perceived decisive advantage that AI-enabled weapons can provide. *Jus ante bellum* therefore requires not only that states not deploy AI-enabled systems without rigorous TEVV, but that they engage in development of these systems in ways that minimize the risk of a security dilemma. The next section discusses what states can do to conduct rigorous TEVV, while the following section discusses how they might take steps to avoid triggering a security dilemma.

## Testing, evaluation, verification, and validation

Deployment of AI-enabled weapons that have not been rigorously tested for safety and reliability would increase the risks of unjust resort to war and harm to innocent persons. To avoid these risks, deployment should be preceded by rigorous engagement in a process known as testing, evaluation, verification, and validation (TEVV). This process, drawn from systems engineering, is designed to assess the future performance of new technology and the risks that it may pose. While TEVV is the most common description of the steps in this process, terminology can vary, and the steps themselves are not strictly separate.

In the defense setting, the Department of Defense Instruction on Test and Evaluation (T&E) says, “The fundamental purpose of T&E is to enable the DoD to acquire systems that support the warfighter in accomplishing their mission” [UD Department of Defense, 2021, §3.1(a)]. Verification seeks to ensure that the technology meets the specifications that a prospective user has provided, while validation assesses whether those specifications will meet the goals of the user (Hand and Khan, 2020).

The TEVV process thus seeks to provide assurance that technology will work as expected, which generates what Roff and Danks call predictability-based trust (Roff and Danks, 2018). Because a weapon can cause significant harm, however, TEVV of weapon systems also must provide what Roff and Danks call values-base trust: confidence that a weapon will operate in a way that is consistent with relevant ethical principles.

As Roff and Danks observe, the challenge is that the paradigm of values-based trust is interpersonal relationships, in which trust reflects confidence that another person will act ethically in unpredictable future situations because we know the values and beliefs that guide them (Roff and Danks, 2018, p. 7). Developing such trust in a machine is much more difficult. Yet the more advanced an AI-enabled weapon system, the more crucial the need to trust that the outputs of its automated components are consistent with ethical principles.

TEVV thus must seek to foster the right kind of calibrated trust in commanders who decide to deploy the weapon system and operators who use it. Trust is *calibrated* when the degree of reliance is appropriate to the system's predictable performance in a particular context (Pinelis, 2021). Trust is of the *right kind* when it is grounded not only in predictability but in confidence that a system will operate in conformity with appropriate ethical values (Roff and Danks, 2018). This can be achieved partly by embedding ethical considerations into the TEVV process and assuring operators and commanders that the legal review and TEVV process not only assures predictable performance, but predictable performance in accord with, for example, *jus in bello* principles.

AI-enabled weapons present distinctive challenges for the TEVV process because of their complexity, opacity, and brittleness. While we cannot discuss all these challenges here, we discuss especially significant ones below, and suggest how TEVV should respond to them in order to satisfy *jus ante bellum*.

## Challenges

Generalizing and extrapolating from test results is especially difficult for many AI-enabled weapon systems because of the exceptional difficulty in anticipating all the conditions under which these weapons will operate. It is true that conventional weapons present a similar obstacle to some extent, since we can test only a fraction of the settings in which a weapon may operate. AI-enabled weapons, however, perform extremely complex tasks, they do so in radically unpredictable environments, and they provide “non-deterministic, dynamic responses to those environments” (Wojton et al., 2021, p. 4). Their likely failures also will be harder to predict and understand than those of conventional weapons. All this makes the range of potential scenarios to test immense, if not infinite.

Compared with conventional weapons, we therefore will be able to generalize with less confidence about performance across varied environments, and less easily identify settings to which the use of a weapon should be confined (Pinelis, 2021). In addition, it may be necessary to move away from insistence on complete risk avoidance and precise risk quantification toward acceptance of some risk of failure. This would involve a focus on ensuring that a system fails “gracefully” in ways that do not cause harm or jeopardize the larger operation in which it is deployed (Pinelis, 2021).

Another challenge is that, while conventional weapons may feature components from several sources, this is especially true of AI-enabled weapons.^4^ This is because much cutting-edge AI development is occurring in the private sector and is being incorporated as components into military systems, and because AI is often utilized to serve specific functions within a larger weapon system. This can make it difficult to assemble large data sets that enable robust tests of all AI-enabled components in a system.

## Adapting TEVV to AI-enabled weapons

Given these ways in which AI-enabled weapons are different from conventional ones, the TEVV process needs to be adapted to address the challenges they present. We focus here on key changes that would serve the requirements of *jus ante bellum* to assure safety, precision and accuracy; to avoid unjust resort to war; and to ensure that AI-enabled weapons do not cause unjustifiable harm.

### TEVV throughout the weapon lifecycle

The TEVV process should be ongoing. TEVV should track the lifecycle of the system, and some aspects of TEVV need to be repeated when the system gets deployed in a new operational environment (Flournoy et al., 2020). As the parameters of this weapon change in response to different features in its environment, it will be necessary to determine when these changes effectively produce a new weapon that requires a new TEVV, or when a new TEVV is necessary for one or more of its components. Furthermore, it will be necessary for a robust TEVV process not to only assess performance in appropriate operational environments, but to define those environments, often in collaboration with those who are developing or integrating AI into weapon systems.

### Training data

Many algorithms relevant to weapon systems, such as object recognition or decision augmentation warfighting algorithms, are trained in simulated environments built on machine-learning algorithms. Simulation-based testing data, however, will be problematic when the risks of deploying a weapon are especially high. In these cases, data sets based on actual conditions are preferable because they can increase commanders' and operators' ability to trust a system in high-risk operational environments.

### Gradual deployment

It will also be crucial in many cases that an AI-enabled weapon be deployed only gradually. “[A] strategy of graded autonomy (slowly stepping up the permitted risks of unsupervised tasks, as with medical residents) and limited capability fielding (only initially certifying and enabling a subset of existing capabilities for fielding) could allow the services to get at least some useful functionality into warfighters' hands while continuing the T&E process for features with a higher evidentiary burden” (Wojton et al., 2021, p. 20).

### TEVV should consider alternatives to use of AI

While TEVV should be adapted to meet the challenges of AI-enabled weapon systems, it also should be used to help identify when using a human, or some other alternative to AI, should be used for one or more components of a system. This would rest upon assessment of how well different systems would achieve the goals of a weapon, taking into account its performance and risks. In other words, TEVV should not simply assess the safety and precision of a weapon in isolation, but should do so in comparison with available alternatives for similar functions in different operational environments.

### TEVV should drive certification schemes

The iterative process used in TEVV can help guide appropriate training, skills and certifications of operators. For example, the US Joint AI Center proposal included four types of testing: algorithmic testing, human-machine testing, systems integration testing, and operational testing with real users in real scenarios (Pinelis, 2021). The human-machine testing and the operational testing provide evidence not just for the evaluation of the weapon, but for how a weapon should incorporate and present machine outputs in order to augment human judgment in the decision-making process in the best possible way. While TEVV has always played a role in US certification schemes for operators, the training content involved in conducting TEVV in certification schemes for AI-enabled weapons may well be significantly greater.

In the ways we have described above, a TEVV process that is sensitive to the challenges of AI-enabled weapons can meet the requirements of the *jus ante bellum*. As the next section discusses, however, this alone will be insufficient to meet these requirements if a state develops these weapons in ways that trigger the security dilemma.

## The security dilemma

The security dilemma exists when one state's investment in military capabilities prompts other states to increase their own investments because they perceive that the first state's actions make them less secure. Two factors may be especially important in triggering this dilemma. The first is when states perceive that the offense has the advantage over the defense, and that they may need to act first to preempt a threat. The second is when it is difficult to distinguish whether a state is developing offensive or defensive weapons, which prevents states from signaling their intentions. Together, these can generate a sense of insecurity that creates incentives for states to develop and deploy weapons as soon as possible. As the discussion below describes, various features of AI-enabled weapons may make these conditions especially likely to occur. The result the risk that states could rush to deploy such weapons without conducting rigorous TEVV.

### AI-fueled security dilemma

First, AI-enabled weapon systems will not be directly observable in the way that conventional weapons are. Whether a system is enabled by AI depends not upon its visible physical characteristics but the software that guides its operation. This means that it is likely to be extremely difficult for one state to determine the AI-enabled weapon capabilities of another.

Second, the dynamic rate of AI innovation means that even if it were possible to make an assessment of a state's AI-enabled capabilities at one point, this assessment may soon be outdated. Third, AI is not itself a weapon but a technology that can be put to a variety of uses. A state therefore faces a considerable challenge in attempting fully to comprehend all the ways in which other states may be incorporating AI into their military operations. Fourth, unlike during the Cold War, states have little experience with the use of AI-enabled weapons that could provide a shared understanding of their capabilities and risks, and thus a basis for negotiating limitations.

Finally, the nature of AI-enabled weapons may intensify a security dilemma because of the perceived decisive advantage of operating at machine speed compared to a “remotely controlled, ‘slower' adversarial system” (Altmann and Sauer, 2017, p. 119). A state may feel especially vulnerable because it fears that another state's use of such weapons against it would inflict grave damage that would prevent it from defending itself or responding. Under these circumstances, states are likely to believe that the balance of military capabilities favors the offense, which can make a preemptive strike seem advantageous.

## Avoiding the security dilemma

What might states do to minimize the risk that development of AI-enabled systems will generate a security dilemma that could risk their harmful deployment? One important measure is to avoid using language likely to trigger a sense of insecurity on the part of other states. A state should avoid characterizing its systems as providing it with an unprecedented decisive military advantage over other states. Language that can create the same risk is the public declaration that states are engaged in an “AI arms race.” Unfortunately, there is no shortage of such language.^5^ Framing the situation in this way suggests that states need to invest in developing and deploying AI-enabled weapons as soon as possible if they want to be secure. A state therefore will need to find a balance between signaling that it has capabilities that should discourage other states from attacking it, while not representing these capabilities as providing it with an overwhelming advantage.

States also can seek to engage in confidence-building measures (CBM) that are designed to reduce states' suspicion of one another through the exchange of information about capabilities and intentions, which may enable some agreement on how operations will be conducted.^6^ Such measures gained particular prominence during the Cold War as a way of reducing the likelihood that misinterpretation of capabilities and intentions could lead to nuclear war.

One measure is for a state to announce publicly that it is committed to ensuring that deployment of these systems is consistent with ethical principles and legal requirements, and that there is assurance of their reliability and safety.^7^ The US Defense Innovation Board, for instance, has released *AI Principles: Recommendations on the Ethical Use of Artificial Intelligence by the Department of Defense*, which have been adopted by the Department (US Department of Defense, 2020). These signal to other states that the US military will develop and deploy AI systems only after careful review to ensure that they can be used ethically. In addition, DoD is adapting both its TEVV and weapons review process to conduct assessments of AI-enabled systems. Publicly committing to these measures can serve as a “costly signal” to other states that they will not be disadvantaged by likewise committing to use AI-enabled weapons only after such review.

A second step could be to work to develop common definitions and shared understanding among states of core concepts that are relevant to the safety, reliability, impact, performance, and risks of AI-enabled weapon systems. A third measure would be to encourage information-sharing and communication channels among states. Some degree of transparency about TEVV, for instance, could involve public release of general information about the process for assessment of military AI-enabled systems without disclosing their specific technical features. This would be similar to the US approach to weapons review, which involves disclosing the process but not the review of particular weapons, in an effort to encourage other states to conduct reviews.

States might also share information on how to establish parameters that limit the domain in which a system can operate without human supervision, and how safely to shut it down if it begins to pose risks by operating beyond that domain. There could be some risk to a state from sharing such information, since it could enhance the ability of adversaries to deploy effective and reliable systems that they could use to threaten the sharing state's security. A state therefore would need to decide how to weigh the security risk of an adversary's improved AI capabilities compared to the risk of an adversary and other states deploying unsafe and unreliable AI systems in ethically problematic ways.

The measures described above could also help build confidence by serving as the impetus for a fourth step, which is establishing common norms and codes of conduct about the deployment and use of AI-enabled systems. Over time, states might bolster these measures by taking a fifth step, which is providing for some degree of inspection and verification. One measure could be for states to share the general characteristics of an AI-enabled weapon without revealing all its training data or other components that they may fear would compromise security. Another might be to permit outside parties to observe the operation of the system without disclosing its algorithms.

Finally, states might work to develop “rules of the road” for the conduct of AI-enabled military operations and perhaps “red lines” that establish limits on their use. States also could agree to declare some geographic areas off limits to autonomous systems because of their risk of unanticipated interactions, as well as pledge not to incorporate AI into their nuclear weapon systems.

## Conclusion

The concept of *jus ante bellum* expands the just war tradition by suggesting that the way in which states prepare for war can be subject to ethical assessment. The distinctive risks of AI-enabled weapon systems make such an assessment especially important. We argue that ethical development of AI-enabled weapon systems requires that a state engage in rigorous testing of a system before its deployment, and that it develop its systems in ways that do not create a security dilemma that would prompt other states to deploy its own systems without such testing. Both steps can be challenging, but they are essential to ensure that weapons are used in ways that are consistent with human values.

## Author contributions

MR took the lead on the section on the security dilemma, while JD did so for the section on testing and evaluation, but each reviewed and helped edit the other's sections. All authors contributed to the article and approved the submitted version.

## Footnotes

^1^Furthermore, while, for example, an autonomous driving tank might be AI-enabled in some sense it raises very different issues than those “AI weapons” that use AI for primarily for war-fighting purposes.

^2^Various sources have the “VV” as “validation and verification” (Flournoy et al., 2020), while NSCAI (2021) and DoD AI strategy documents have it as “verification and validation.” Here we use VV to mean verification and validation, partly because we see sources such as NSCAI as authoritative in the U.S. context, and partly because validation is the last step in the process in which machine-learning models are built and tested (We thank Joe Chapa for this clarification).

^3^Careful TEVV can uncover such explainability limitations and that can in turn inform potential remedies to inscrutability; remedies that might include re-training of operators, changing the user interface, augmenting the algorithm with XAI tools, or in some cases when such lack of explainability significantly negatively affects calibrated trust in operators, abandoning the algorithm.

^4^Interview with Joe Chapa, Chief Responsible AI Ethics Officer for the Air Force.

^5^See, e.g., Geist (2016) and Rickli (2017).

^6^Among the sources on this subject are Desjardin (2014), Bode and Huelss (2018), Horowitz (2018), Imbrie and Kania (2019), Horowitz et al. (2020), Horowitz and Scharre (2021) and Scharre (2021).

^7^It is important to acknowledge that in considering the security dilemma we acknowledge that peer and competitor states might find both generic AI and weapons-specific AI as threatening, but that weapons-specific AI might raise further worries than broad-use AI. The governance of both AI-enabled weapon systems and broad-use AI thus matters for the security dilemma. For governance of broad-use AI, see, for example, White House (2022).
